# Supplementary material for: A single-cell survey of Drosophila blood
Source: eLife. 2020 May 12;9:e54818. doi: 10.7554/eLife.54818 (PMC7237219; doi:10.7554/eLife.54818)
Supplement: Transparent reporting form [file elife-54818-transrepform.docx]

***eLife’s* transparent reporting form**

We encourage authors to provide detailed information *within their submission* to facilitate the interpretation and replication of experiments. Authors can upload supporting documentation to indicate the use of appropriate reporting guidelines for health-related research (see [EQUATOR Network](http://www.equator-network.org/%20)), life science research (see the [BioSharing Information Resource](https://biosharing.org/)), or the [ARRIVE guidelines](http://www.plosbiology.org/article/info:doi/10.1371/journal.pbio.1000412) for reporting work involving animal research. Where applicable, authors should refer to any relevant reporting standards documents in this form.

If you have any questions, please consult our Journal Policies and/or contact us: [editorial@elifesciences.org](mailto:editorial@elifesciences.org).

**Sample-size estimation**

- You should state whether an appropriate sample size was computed when the study was being designed
- You should state the statistical method of sample size computation and any required assumptions
- If no explicit power analysis was used, you should describe how you decided what sample (replicate) size (number) to use

Please outline where this information can be found within the submission (e.g., sections or figure legends), or explain why this information doesn’t apply to your submission:

For all the scRNA-seq analysis (Figures 1-5), we did not use any sample size computation nor power analysis. We decided to have at least **3-4 independent biological replicates per condition** and sufficient number of cells (**~19K**) in total. This information can be found in the methods and results sections.

**Replicates**

- You should report how often each experiment was performed
- You should include a definition of biological versus technical replication
- The data obtained should be provided and sufficient information should be provided to indicate the number of independent biological and/or technical replicates
- If you encountered any outliers, you should describe how these were handled
- Criteria for exclusion/inclusion of data should be clearly stated
- High-throughput sequence data should be uploaded before submission, with a private link for reviewers provided (these are available from both GEO and ArrayExpress)

Please outline where this information can be found within the submission (e.g., sections or figure legends), or explain why this information doesn’t apply to your submission:

As indicated in methods and results sections pertaining to each figure, the scRNA-seq analysis was performed with 3-4 biological replicates per condition (Fig. 1).

For the Monocle 3 (Figure 3), we used cells from 2 biological replicates each of the unwounded and wounded datasets to avoid batch effects coming from various technologies that may affect the pseudotime analysis. **This information can be found in results and methods section.**

Confocal imaging in Figure 4: 5, 6, and 12 independent biological replicates (larvae) were used in a given experimental day with a total of three independent times to achieve a total of n=23 independent biological replicates. **This information can be found in the Fig. 4G legend.**

Confocal imaging in Figure 5: 3 independent biological replicates (n=3) were used. **This information can be found in the Fig. 5G legend.**

Validation of Bnl, Btl in Figure 6: 20 independent biological replicates (n=20) for Bnl validation. **This information can be found in results section.**

Bleeding and counting the Btl+ LMs was done with 5 independent biological replicates (n=5). **This information can be found in results section.**

The information related to melanization frequencies in larvae can be found in the results section.

**Statistical reporting**

- Statistical analysis methods should be described and justified
- Raw data should be presented in figures whenever informative to do so (typically when N per group is less than 10)
- For each experiment, you should identify the statistical tests used, exact values of N, definitions of center, methods of multiple test correction, and dispersion and precision measures (e.g., mean, median, SD, SEM, confidence intervals; and, for the major substantive results, a measure of effect size (e.g., Pearson's r, Cohen's d)
- Report exact p-values wherever possible alongside the summary statistics and 95% confidence intervals. These should be reported for all key questions and not only when the p-value is less than 0.05.

Please outline where this information can be found within the submission (e.g., sections or figure legends), or explain why this information doesn’t apply to your submission:

All the statistics for the replicates mentioned above were performed using Prism 8. The “Statistics” section in Methods describes the statistical methods used and the detailed information with regards to the t-test, One-way ANOVA, and the error bars (SEM or SD) can be found in the figure legends wherever necessary. The exact p values are also mentioned alongside in the figures and figure legends, wherever necessary.

(For large datasets, or papers with a very large number of statistical tests, you may upload a single table file with tests, Ns, etc., with reference to sections in the manuscript.)

**Group allocation**

- Indicate how samples were allocated into experimental groups (in the case of clinical studies, please specify allocation to treatment method); if randomization was used, please also state if restricted randomization was applied
- Indicate if masking was used during group allocation, data collection and/or data analysis

Please outline where this information can be found within the submission (e.g., sections or figure legends), or explain why this information doesn’t apply to your submission:

N.A.

**Additional data files (“source data”)**

- We encourage you to upload relevant additional data files, such as numerical data that are represented as a graph in a figure, or as a summary table
- Where provided, these should be in the most useful format, and they can be uploaded as “Source data” files linked to a main figure or table
- Include model definition files including the full list of parameters used
- Include code used for data analysis (e.g., R, MatLab)
- Avoid stating that data files are “available upon request”

Please indicate the figures or tables for which source data files have been provided:

All tables are provided as Supplementary tables.

Source data files are provided to support the bar graphs in Figures 2D, 4B, and 5B.

Source data files is provided to support the scatter plot in Figure 4G.

Source data file is provided to support the bar graph in Figure 5I.
